# Desired and Undesired Effects of Energy Labels—An Eye-Tracking Study

**DOI:** 10.1371/journal.pone.0134132

**Published:** 2015-07-31

**Authors:** Signe Waechter, Bernadette Sütterlin, Michael Siegrist

**Affiliations:** Institute for Environmental Decisions (IED), Consumer Behavior, ETH Zurich, Zurich, Switzerland; University of Pécs Medical School, HUNGARY

## Abstract

Saving energy is an important pillar for the mitigation of climate change. Electric devices (e.g., freezer and television) are an important player in the residential sector in the final demand for energy. Consumers’ purchase decisions are therefore crucial to successfully reach the energy-efficiency goals. Putting energy labels on products is often considered an adequate way of empowering consumers to make informed purchase decisions. Consequently, this approach should contribute to reducing overall energy consumption. The effectiveness of its measurement depends on consumers’ use and interpretation of the information provided. Despite advances in energy efficiency and a mandatory labeling policy, final energy consumption per capita is in many countries still increasing. This paper provides a systematic analysis of consumers’ reactions to one of the most widely used eco-labels, the European Union (EU) energy label, by using eye-tracking methodology as an objective measurement. The study’s results partially support the EU’s mandatory policy, showing that the energy label triggers attention toward energy information in general. However, the energy label’s effect on consumers’ actual product choices seems to be rather low. The study’s results show that the currently used presentation format on the label is insufficient. The findings suggest that it does not facilitate the integration of energy-related information. Furthermore, the current format can attract consumers to focus more on energy-efficiency information, leading them to disregard information about actual energy consumption. As a result, the final energy consumption may increase because excellent ratings on energy efficiency (e.g., A^++^) do not automatically imply little consumption. Finally, implications for policymakers and suggestions for further research are discussed.

## Introduction

Reducing energy consumption is a declared goal in many countries (e.g., [1]). Important reasons for decreasing energy use include economic and ecological benefits. Moreover, reducing energy plays an important role in mitigating climate change. For example, less energy consumption can help reduce carbon emissions, requiring fewer power plants now and in the future (e.g., [2, 3]). Considering the fast growth of the global population, this undertaking is as important as it is challenging. Households are responsible for approximately 30% of the final energy consumption [4–6]. In private households, about 70% of energy is used for heating, approximately 15% for warm water, and about 12% for household appliances and consumer electronics such as televisions, computers, refrigerators, and freezers [7, 8]. Consequently, households or more precisely, consumers constitute a segment that needs to be addressed to reach energy-saving goals. In many countries, the reduction of energy consumption is tackled by trying to enhance energy efficiency [9, 10]. For example, the European Union (EU) plans to save up to 20% of its members’ energy consumption by 2020, mostly by increasing energy efficiency [9, 11]. Hence, the EU released minimum standards regarding energy efficiency in several domains, such as buildings, household appliances, and consumer electronics. Consequently, new products on the market have to fulfill these requirements; the sale of energy-inefficient products is restricted as well [12].

**Fig 1:**
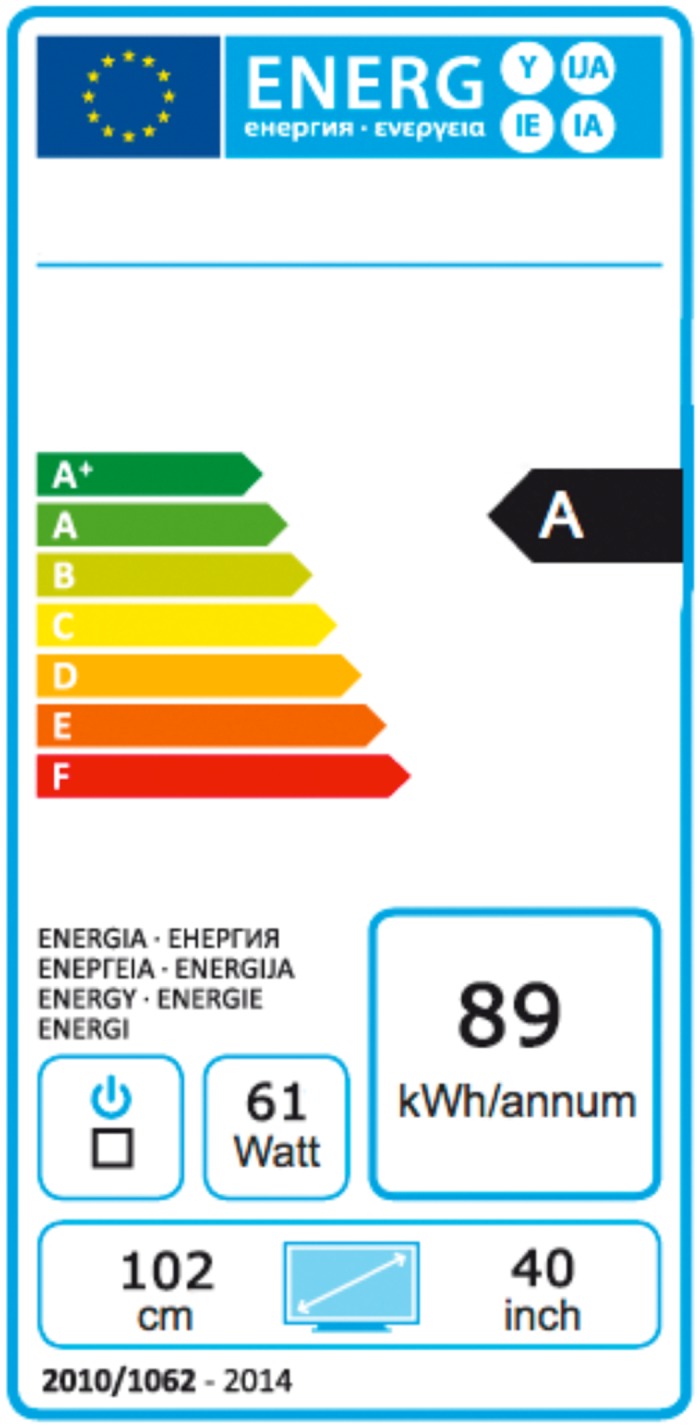
EU energy label used for televisions.

In 1992, the Council of the European Communities introduced an energy label to target consumers’ decision making at the point of sale [13]. The energy label should facilitate an energy-friendly choice of electric goods. The energy label provides two sources of information—energy efficiency and actual energy consumption—to assess the energy friendliness of an appliance. Information about a product’s energy efficiency is communicated with its letter rating on a scale and its place on a certain spectrum of color codes. The letter scale originally ranged from A to G, with A as the most efficient and G as the least efficient products. However, the rapid development of highly energy-efficient items and the ban on inefficient ones on the market required the introduction of new rating classes to differentiate among products with the best (A) rating on energy efficiency. Therefore, plus markers (e.g., A^+^) were also implemented [9, 11]. For some products, the energy-efficiency rating now ranges from A^+++^ to D (e.g., freezer), whereas for others, it encompasses A^+^ to F (e.g., television) or continues as simply A to G (e.g., coffee machines). An additional cue for a product’s energy efficiency is the color code that displays its rating (Fig 1). The color code ranges from red to green, where red represents poor performance in terms of energy efficiency, while green signifies excellent performance.

The energy-efficiency letter rating reflects the power consumption of a product, based on its size. For example, a television’s energy efficiency is basically calculated by the power consumption (watt) per square decimeter (dm^2^) of the visible screen. According to this performance the products are assigned a letter ranking. This means that both a small and a large television can have the same energy efficiency rating (e.g., A), because per dm^2^ their energy consumption levels are equal. However, the actual energy consumption levels are different, because they differ in size (i.e., the larger television has a greater dm^2^). Thus, for consumers it is not self-explanatory how the letter categorization system of the energy-efficiency rating (e.g., A^+^) reflects this relative calculation. Hence, this classification should not be used to compare different-sized products, such as a 50-inch against a 60-inch television, to assess their energy friendliness (i.e., find the product with the lowest consumption). The information about a product’s actual energy consumption is communicated in kilowatt-hours per year (e.g., 100 kwh/annum). Thus, other than the energy-efficiency rating, annual consumption is an absolute numerical value that allows comparison among differently sized products.

The energy label is mandatory for a wide range of electric devices, including household appliances (e.g., refrigerator and dryer) and consumer electronics (e.g., television) and it has to be placed on the products sold in stores. Furthermore, the energy-label requirement is constantly broadened to new product types. It constitutes one of the most important policy tools of the EU to reach the targeted energy goals, namely the reduction of energy consumption by increasing energy efficiency [9, 11]. The energy efficiency of household appliances (e.g., washing machines and freezers) and consumer electronics (e.g., televisions and laptops) has been improving since the introduction of the energy label in 1992. However, the actual residential electricity consumption had been increasing by 2% per year from 2001 to 2011 [14]. This trend can partially be explained by a higher level of amenities, a general enhancement of basic comfort and population growth [4, 14]. Although some European countries (e.g., the United Kingdom) managed to substantially decrease their consumption per capita over the past years, overall for the EU’s 27 member states, the final consumption only shrank very little, and many countries even increased their energy consumption [5]. Trend observations by the World Bank have shown that this effect also holds true for the rest of the world [15, 16]. Of special concern is that the Organisation for Economic Cooperation and Development (OECD) countries still account for 65% of the residential electricity consumption worldwide [17]. This means that the increasing trend of energy consumption cannot be explained by the development of non-OECD countries. Moreover, information and communication technologies and consumer electronics have been identified as the most quickly growing sector in terms of final electricity consumption [17]. To successfully reduce energy consumption, it is therefore important to investigate the reasons for the undesirable increase in energy consumption. Previous evaluations of actual energy use have already revealed an energy-efficiency gap, pointing out the difference between actual and estimated potential energy savings [18]. Allcott and Greenstone recently referred to this gap as “investment inefficiencies […]: a wedge between the most-minimizing level of energy efficiency and the level actually realized” ([19], p. 134). Experts disagree about the magnitude of the energy-efficiency gap because most estimates do not rely on randomized controlled trials but on engineering analysis and many interventions are not as energy saving as estimated by technicians [19, 20]. However, according to the International Energy Agency (IEA), switching to the most efficient products could save about 40% of residential electricity consumption [17]. Gillingham and Palmer [21] concluded in a review article that behavioral effects or more precisely, consumers’ purchase decisions constitute one reason for the energy-efficiency gap. This conclusion stands in contrast to findings of recent studies that highlighted the relevance of energy efficiency to consumers [22–24]. For example, a study involving German television users found that willingness to pay increased with higher energy efficiency [24]. A study in the United States showed that consumers indicated a higher willingness to pay for products labeled with the Energy Star [25]. Therefore, the question is raised why there is a mismatch between consumer statements and actual energy consumption. Expressed differently, what psychological effects might impede energy savings resulting from energy-efficiency measures?

Recent research revealed that the energy-efficiency gap was aggravated by insufficient implementation of the EU policy on the energy label (e.g., not placed on products in stores) and institutional problems, such as weak support by different stakeholders (e.g., nongovernment organizations) [26, 27]. Additionally, the energy label is not yet mandatory for online shops that are gaining market share [28]. Other developments indicate undesirable consumer behavior, such as the observed trend toward larger appliances [6, 26]. This trend suggests that it may be essential to consider psychological side effects triggered by the energy label and the promotion of energy efficiency (e.g., [29–32]). A recent study found evidence for consumers’ misinterpretation of energy efficiency showing their tendency to focus excessively on energy-efficiency information and to neglect actual energy consumption when making estimates of a product’s energy friendliness [31]. This study indicated the participants’ susceptibility to the so-called energy-efficiency fallacy. This fallacy refers to people’s tendency to assess a product’s performance in terms of energy consumption based on its energy-efficiency rating. This derivation is problematic, as explained in the previous section, because the energy efficiency rating on the energy label only provides a suitable basis for comparison with similar products (i.e., products of the same category *and* size). However, if two products differ in size, the energy-efficiency rating does not provide an adequate information basis for selecting the product with less energy consumption. Moreover, the study by Waechter and colleagues [31] had detected that excellent energy-efficiency ratings (e.g., A^+++^) could even distort the perception of entire product categories. This means that consumers’ perception of product categories that are generally associated with high energy consumption (i.e., freezers) can shift to energy friendliness due to people’s reliance on energy-efficiency information, although the actual consumption of such products is still high. This energy-efficiency fallacy is a matter of concern because the promotion of energy efficiency constitutes the core of energy strategies in various countries [9, 33, 34]. Similar concerns can be found in the literature, criticizing the policy to concentrate merely on the promotion of energy efficiency [35–37]. It has been argued that the promotion of energy efficiency and the energy-efficiency rating on the energy label critically neglect the role of actual energy consumption (e.g., [12, 17, 38]). Thus, the energy label may in fact enhance energy consumption by misleading consumers to overestimate the role of energy efficiency. Based on these theoretical and empirical considerations, it seems questionable whether the energy label causes the desired effects regarding consumers’ decision making. In other words, what is the energy label’s performance level concerning consumers’ energy-friendly decision making and purchase behaviors?

### Consumers’ Decision-making Process

Consumers are confronted with a wide range of information at the point of sale. Ideally, they evaluate all information provided and make an informed decision. However, research on decision making suggests that this ideal behavior is not what can commonly be expected. On the contrary, people tend to rely on cognitive shortcuts, such as heuristic strategies, to reach a decision (e.g., [39, 40]). People often use heuristics when they want to avoid cognitive effort. Heuristic processes can be conscious or unconscious, but what they all have in common is ignoring part of the information [41]. For information processing, this means that consumers do not integrate all the information provided on products but base their decisions on a limited number of information cues.

The presentation format of information is crucial in determining whether or not a certain cue is evaluated. More precisely, information that is presented in a salient and accessible format is more likely to be integrated compared to information that is more complex or not prominently presented [42]. The second type of information is therefore often less influential in the decision-making process. For example, a study by Schulte-Mecklenbeck and colleagues [43] investigating consumers’ food choices revealed that participants chose a meal mainly based on how appealing it looked on a picture (i.e., easily accessible), not based on nutritional values (i.e., complex information). Hence, product labeling seems an adequate way to reach consumers because information can be presented in a noticeable and accessible format (e.g., use of colors and pictograms). For example, a study by Siegrist and colleagues [44] examining different nutrition labels showed that the traffic light system helped consumers process information efficiently and quickly. The same was true for the effect of the Energy Star label, which allowed participants to quickly derive a product’s energy friendliness [25]. Consequently, the use of labels is perceived as an adequate way to inform consumers and to evoke awareness of the label’s objectives. It is often claimed that one benefit of labels is that they convey information in an easily accessible format and can help to close a possible information gap (e.g., [44–46]). Therefore, our hypotheses regarding the energy label’s benefits were the following:

Hypothesis 1: Presenting consumers with the energy label influences how intensively (i.e., how long) they focus on energy-related information.

Hypothesis 2: The integration of energy-related information is easier (i.e., less time is needed to process the information) if the energy label is available as an additional source of information.

Hypothesis 3: The presentation of the energy label alters consumers’ product choices.

However, some studies suggested that labels could lead to imperfect communication because consumers failed to grasp the detailed meaning of some cues [27, 47, 48] and simply used the labels as signs of approval, instead of an actual source of information (e.g., [49, 50]). For example, a meta-study of eco-labeling systems in the United States [46] showed that those that functioned as seals of approval (e.g., Energy Star) were preferred by consumers. The authors concluded that only a few consumers were willing and able to use and interpret technical information provided on labels.

This issue highlights an important drawback of fast and frugal decision-making strategies. Such heuristics are efficient as long as the information that serves as a basis for the decision is precise and not contradictory [41]. If this is not the case, heuristic decision making can lead to the neglect of important information (e.g., [51]) and result in biased decisions and misjudgments. Based on previous literature, it is known that the salience, accessibility, or symbolic meaning of cues can bias information search and decision making (e.g., [40, 52, 53]). For example, the affect heuristic [40] states that salient and accessible cues are easily mapped into an affective response (e.g., green energy-efficiency rating) compared with ambiguous information cues, such as technical or numerical information (e.g., kilowatt-hours). Consequently, the cues linked to a stronger affect receive more weight in the decision making and substitute for the less accessible cues [41]. The influence of symbolically significant information was shown in a study by Sütterlin and Siegrist [52]. People relied on the symbolically significant information (i.e., the car type—driving a Prius vs. driving a sport utility vehicle [SUV]) when they were asked to judge the environmental impact of driving behaviors. Other relevant information with less symbolic significance (e.g., actual distances covered) was largely ignored. As a result, people judged the Prius driver’s behavior as more energy friendly than that of the SUV driver, although the latter actually covered a much shorter distance and therefore used less fuel (i.e., more environment-friendly behavior).

These findings also have implications for the promotion of energy-friendly consumer behavior. Regarding the EU energy label, information about energy efficiency (e.g., A) that is communicated with a single letter and a prominent color code is more easily accessible and may represent a stronger symbolic meaning than the numerical information format of actual electricity consumption (e.g., 50 kwh/annum). Several studies have shown that consumers often have difficulties in understanding information about actual electricity consumption (e.g., expressed in kilowatt-hours per year) and deciding whether a certain energy consumption is high or low (e.g., [54, 55]). Furthermore, information about the actual electricity consumption of devices is rather unimportant for consumers’ decisions [56]. The current presentation format of energy-efficiency information on the energy label may therefore be a potential trigger for heuristic thinking processes and can lead to the disregard for important information, such as the actual electricity consumption. Consequently, consumers may choose efficient products that still consume a considerable amount of energy, based on the mistaken notion that energy efficiency implies low energy consumption. Thus, understanding consumers’ information-processing and decision-making strategies is necessary in order to assess the effectiveness of policy tools, such as the EU energy label. We therefore formulated the following hypotheses regarding consumers’ evaluation of the information on the energy label:

Hypothesis 4: The presentation of the energy label guides consumers to focus more often on energy-efficiency information and less on actual energy consumption.

Hypothesis 5: Integration of energy-efficiency information is easier (i.e., less time is needed to process the information) than that of actual energy consumption.

### Consumers’ Product Choices

Product choice is strongly influenced by personal preferences, for instance, regarding product brand, price sensitivity, and space restrictions. To account for this effect in choice tasks, we introduced two different treatments––a self-focus condition where participants were asked to choose for themselves and an energy-saving focus where participants were asked to choose a product for a person who would want to use as little energy as possible. The self-focus condition of the factor focus thereby corresponded to a realistic purchase situation and allowed the assessment of the energy label’s influence on information search behavior in a free-choice setting. This means that participants were expected to choose and to evaluate the information provided according to their own individual preferences (e.g., price, size, and design). To understand the evaluation of energy-related information and to have a condition without the influence of personal preferences, the second condition with the energy-saving focus was included. In this condition, participants were expected to ignore personal preferences and to decide based on energy-related information in order to recommend the most energy-friendly product. The goal was to understand consumers’ use and interpretation of energy-related information and to assess a possible impact of the energy label on this behavior. Regarding the effect of the focus condition, we formulated the following hypothesis:

Hypothesis 6: The focus on energy-related information is higher in the energy-saving focus condition compared to the self-focus condition.

Furthermore, the energy-saving focus condition would allow testing the degree of complexity of the different energy-related information.

### Methodological Approach

To address the research questions, two hypothetical choice experiments for two consumer products (televisions and freezers) were designed. Data for the study were collected through eye tracking to gain an objective understanding of consumers’ information search behavior.

In the past years, eye tracking has regularly been used to study consumer behavior (for an overview, see [57]). This methodology provides an objective measurement to understand better how consumers process information, such as product labels. For example, eye tracking has been successfully used to evaluate the effectiveness and perception of nutritional labels (e.g., [44, 58, 59]) and is a promising method to detect and improve knowledge of decision-making strategies (e.g., [60]). However, this study is the first eye-tracking approach investigating the impact of the EU energy label. Based on the eye-tracking data, this paper unveils the influence of the energy label on consumers’ information search behavior. It shows that the energy label can lead to misperceptions and unwanted effects that may potentially impede energy-saving goals. Furthermore, it provides important implications for policymakers and further research.

## Materials and Methods

### Ethic Statements

This study complies with all current laws and regulations of Switzerland, and the ethical review committee of the ETH Zurich approved all procedures.

### Participants

An invitation letter was sent to a random sample of 500 households in the German-speaking part of Switzerland, drawn from the electronic telephone directory. The letter briefly explained the study’s objectives and procedure and announced a follow-up phone call over the next few days to ask about their interest to participate. Additionally, participants were recruited via a free advertisement on a newsletter. The exclusion criteria for participation included ages younger than 20 and over 65 years, wearing eyeglasses or hard contact lenses, or suffering from eye diseases. For eye-tracking studies, participants should not be over 65 years old because aging tends to cause drooping eyelids, which hinder good calibration [61]. Participants with eyeglasses were also excluded because small scratches and/or reflections on the glasses pose a problem for the eye tracker. Furthermore, eye diseases such as cataracts can lead to calibration problems. The experiment lasted around 45 minutes; the participants received CHF 40 (≈USD 42) as an incentive.

In total, 123 people from the population of the German-speaking part of Switzerland agreed to participate in the experiment. Due to incompatibility issues between six participants and the eye tracker, they had to be excluded. This led to a final sample of 63 women and 54 men (N = 117). The mean age was 36 years (SD = 11). The majority of the participants (55.6%) had at least completed high school. One participant reported having a slight case of strabismus. However, since the calibration was good and the tracking ratio was high (i.e., percentage of non-zero gaze positions divided by sampling frequency and multiplied by run duration), the person was not excluded from the study [61]. All participants had normal or corrected-to-normal eye vision.

### Stimuli for the Eye-tracking Experiment

To investigate the impact of the energy label on consumers, we chose two product categories that participants would be familiar with: freezers and television. The television was included because it is frequently bought and used by the general population. On the other hand, the freezer was selected as a stereotype for excessive consumption of products. The two items also differed regarding emotional involvement. It seemed plausible to assume that a television would evoke more interest and technical affinities, whereas in most cases, a freezer would merely represent a cooling unit for food storage. Thus, by including a typical household appliance and a typical consumer electronics product, more general conclusions about the importance of product-specific evaluations of the energy label could be drawn from the results

**Fig 2:**
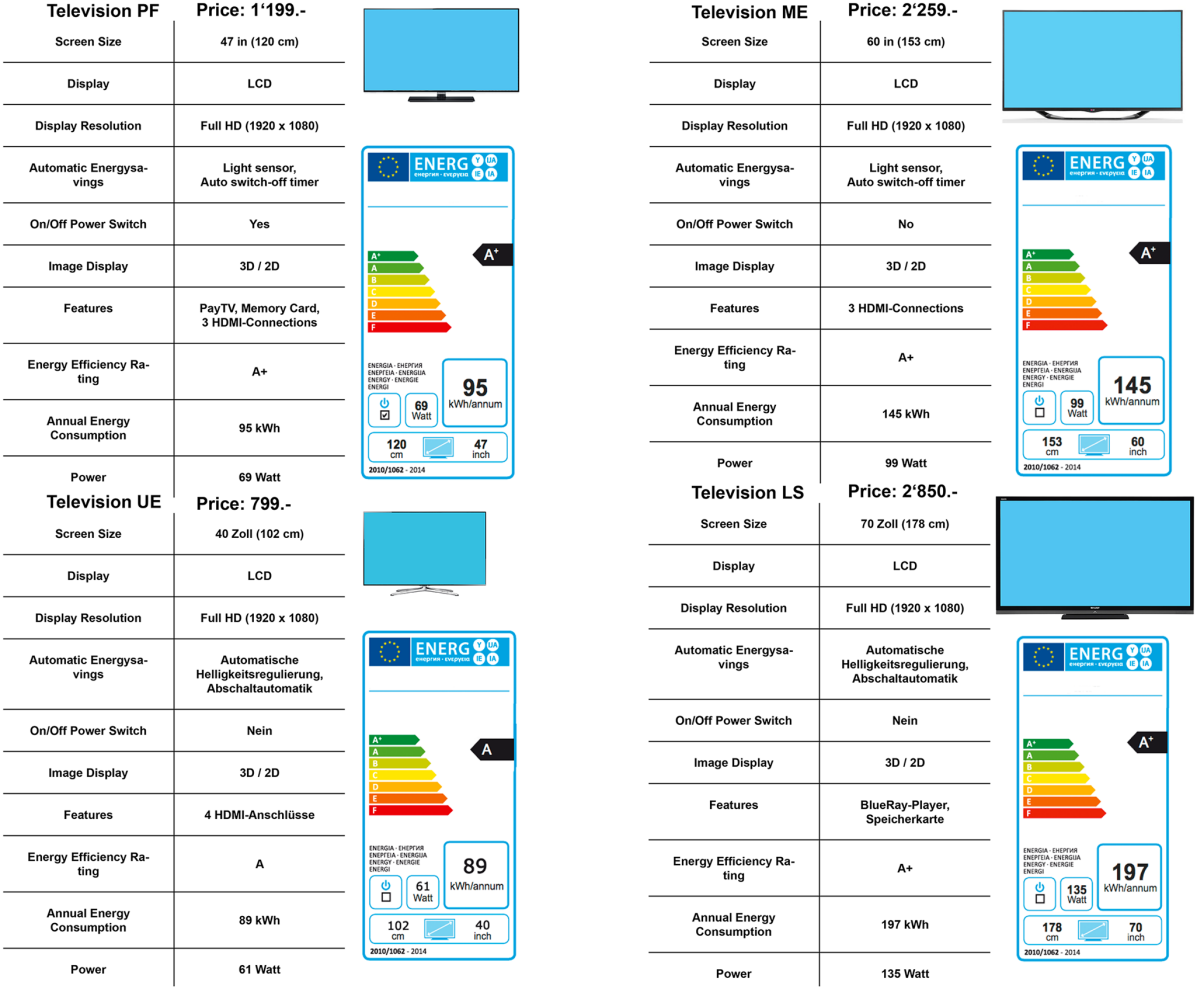
Products with pictures and television features in the label condition. The participants were asked to choose a product either for themselves or for a person who would want to use the least possible amount of energy.

**Fig 3:**
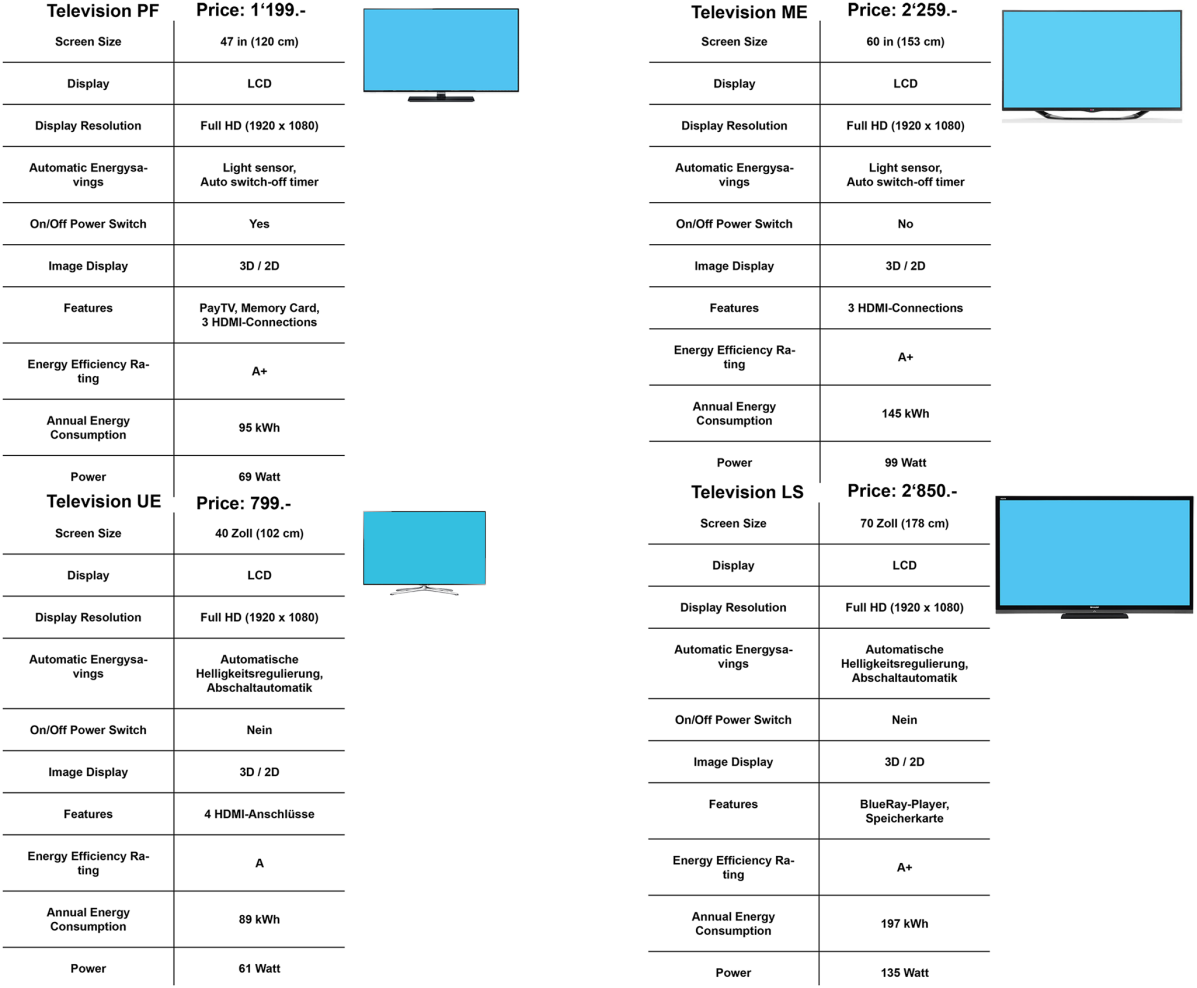
Products with pictures and television features in the no-label condition. The participants were asked to choose a product either for themselves or for a person who would want to use the least possible amount of energy.

The stimuli materials consisted of the descriptions of four models per product category, including pictures of these products, prices, and additional information usually provided in stores (freezer: e.g., volume capacity, energy efficiency, and type of compartments; television: screen size, wattage, and technical features). The chosen products varied regarding energy efficiency, energy consumption, price, size/volume, and technical features. The products represented a selection as could be found in an online shop. The most energy-efficient product was not automatically the most energy-friendly one. The four products were presented simultaneously on one page (Figs 2 and 3; S1 and S2 Figs), and the participants were asked to choose one of the products.

### Experimental Design and Procedure

The iViewX RED500 eye tracker (SMI, Germany) was used. This system provides a binocular sampling rate of 50Hz and an accuracy of 0.4°. Participants’ eye movements are observed with an infrared-sensitive video camera placed below the computer monitor. Specialized software generates x- and y-coordinates for the gaze point on the monitor screen. Experiment Centre 3.3, an application provided by SMI, was used to design and run the experiment.

All participants first read and signed a consent form, acknowledging that their gaze behavior would be recorded, their data would be treated anonymously, and they could quit the study at any time without providing a reason.

To provide good data quality, the eye tracker needed to be calibrated for each subject. The participants were seated in front of the eye tracker at a distance of approximately 70 centimeters with a visual angle of approximately 2 Degrees. The master computer was placed on a second desk, approximately 1.5 meters away from the one with the eye tracker. The examiner explained the device and the calibration to the participants and verified that they had understood the procedure. To minimize body movements, the participants were instructed to place their elbows on the table and to rest their chins in their hands. However, slight head movements to the right or the left would not affect data quality. When the chosen position was comfortable for the participants, the examiner started the calibration on the master computer. The calibration with a deviation of y < 1.5° and x < 1.5° was accepted [61]. The calibration was repeated up to four times per task. The participants were then reminded to remain in their position and to keep their head movements to a minimum.

Subsequently, the instruction for the first task was shown on the screen, and when the participants confirmed that they had read the instruction and had no further questions, the examiner activated the next page with the four products that participants had to view in order to make a choice. When the participants articulated their choice (i.e., by saying the name of the selected product), the examiner immediately pressed the space button, and a blank page followed. By pressing the space button, a time stamp was taken, which could afterwards be used as a measurement of the time that the participants needed for the decision. The examiner noted the participants’ respective choices. Before the second task started, the system was recalibrated. The procedure for the second task was identical to that of the first one, except that the participants now had to choose among four models of another product category (i.e., freezers in the first task and televisions in the second task or vice versa). After the second task, the examiner asked a few qualitative questions about the decision-making process. The qualitative questions were used to gain additional insights into the participants’ information search and decision-making behavior, complementary to the eye-tracking data. The questions were semi-standardized, and the qualitative part lasted for around 5 minutes. They were exploratory in nature and not systematically analyzed for this study. Finally, the participants were asked to fill out a paper-and-pencil questionnaire assessing their sociodemographic information. There were no time restrictions for the experiment.

We used a 2x2 between-subjects design with the factor choice-focus (choice for oneself [self-focus] vs. choice for a person who would want to save energy [energy-saving focus]) and the factor label (label vs. no label). This procedure resulted in four experimental conditions: (1) choosing a product for oneself, with information in a table format (without energy labels); (2) choosing a product for oneself, with information in a table format and the corresponding energy labels; (3) choosing a product for a person who would want to save energy, with information in a table format (without energy labels); and (4) choosing a product for a person who would want to save energy, with information in a table format and the corresponding energy labels. Except for including or excluding the energy labels, the stimuli materials were identical for all four conditions.

The factor focus was only relevant for the task instruction informing the participants that they would see four products, from which they had to choose one (for themselves or for another person). Table 1 presents the instructions for the conditions of the factor focus for the television task. The instruction for the freezers was identical, except that “television” was replaced with “freezer.” The participants were first asked to choose a freezer and subsequently a television or vice versa. The presentation order of the categories changed randomly among the subjects to control for possible order effects. The factor levels (i.e., factor label: label vs. no label; factor focus: self vs. energy saving) did not change during the experiment.

**Table 1:** Task Instructions.

Focus	Instruction
Self	Please imagine that you would like to buy a new television. On the next page, you will be presented with four televisions, out of which you can choose one. Look at the pictures and the information as you would at home on your computer screen. Please decide which television you would buy. Tell the examiner the name of the chosen product. The name is shown on the top left of each product and consists of two characters (e.g., SZ). Take as much time as you need. Please look at the screen during the whole time and try to sit very still. If you have any questions regarding the task, please ask the examiner. If you do not have further questions, inform the examiner that you understood the task and she/he will activate the next page.
Energy saving	Please imagine that a person who wants to use the least possible amount of energy would like to buy a new television. Four televisions are the choices and the person asks for your advice. On the next page, you will be presented with the four televisions, out of which you should choose one. Look at the pictures and the information as you would at home on your computer screen. Please decide which television you would recommend to the person. Tell the examiner the name of the chosen product. The name is shown on the top left of each product and consists of two characters (e.g., SZ). Take as much time as you need. Please look at the screen during the whole time and try to sit very still. If you have any questions regarding the task, please ask the examiner. If you do not have further questions, inform the examiner that you understood the task and she/he will activate the next page.

*Note*. Instructions Used for the Television Choice Task: with self-focus (i.e., hypothetical purchase for oneself) and energy-saving focus (i.e., hypothetical recommendation to a person who would want to save energy).

### Eye-tracking measures

**Fig 4:**
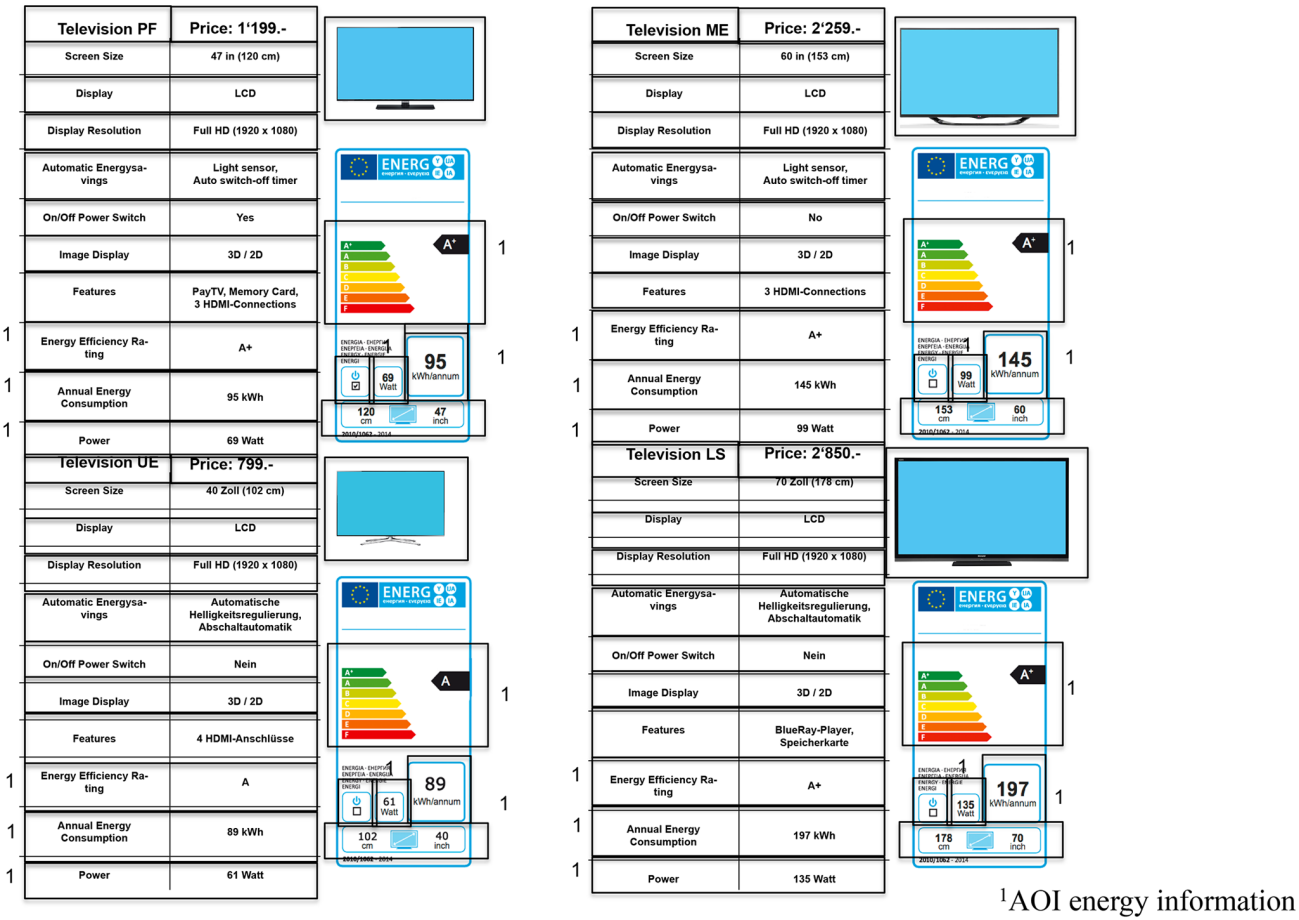
Areas of interest (AOI) defined for the television task in the label condition. Each box represents an AOI used for data analysis. The AOIs marked with number 1 were combined with the AOI energy information.

**Fig 5:**
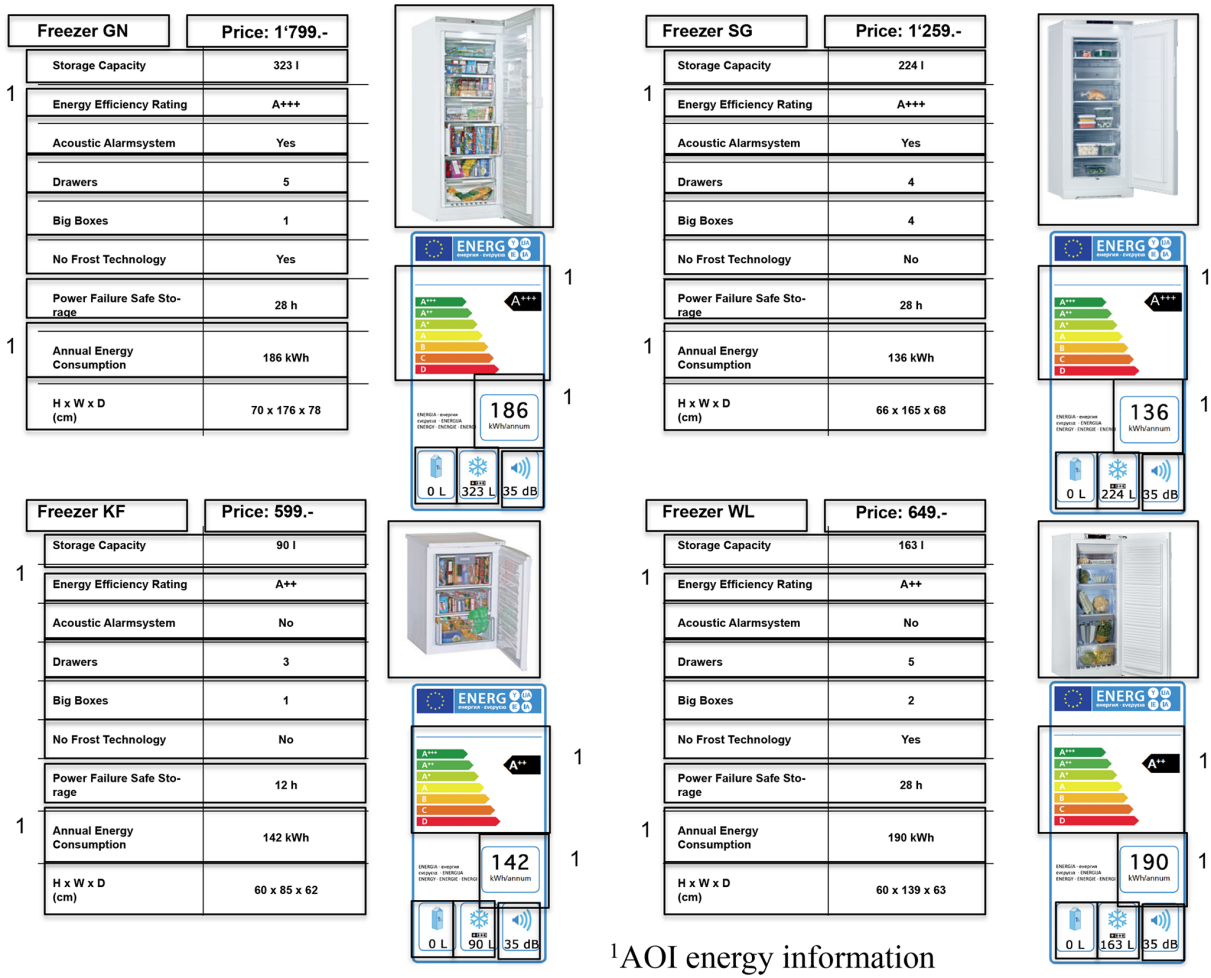
Areas of interest (AOI) defined for the freezer task in the label condition. Each box represents an AOI used for data analysis. The AOIs marked with number 1 were combined with the AOI energy information.

The raw data of the eye tracker was imported into BeGaze (SMI, BeGaze 3.3) for data analysis. In eye-tracking research, two eye movements are mostly of interest: fixations and saccades (e.g., [44, 62, 63]). A fixation is measured when the eye remains still for a certain time period, whereas saccades describe the eye’s rapid movements from one fixation to another. We used the default event detection algorithm provided by the eye tracking software. The parameters for fixation detection are defined with a minimal duration of 80ms and a maximal dispersion of 100 pixels. According to the eye-mind hypothesis, fixations reflect cognitive processes [64]. This means that what we look at is also what we pay attention to in most cases (e.g., [61, 65, 66]). For further analysis, areas of interest (AOIs) were defined for each item presented to the participants (Figs 4 and 5). To assess the participants’ evaluation of the information presented, three parameters within the defined AOIs were derived for data analysis: mean fixation durations, dwell times, and number of fixations. The space outside the AOIs (i.e., whitespace) was excluded for the analysis [61]. Only few outliers and extreme scores were identified in the data. We did not exclude any outliers, but if necessary, an adequate transformation was applied and used for the analysis.

#### Dwell time

Sometimes called gaze duration, dwell time is calculated by summing up all fixations and saccades that hit a particular AOI (i.e., time the gaze stayed on an AOI). It is an indicator of the attention distribution over the different AOIs. Longer dwell times reflect deeper information processing [67]. They are also associated with interest and informativeness [68]; people tend to gaze more often at data that is more important and interesting to them [57]. Thus, this parameter was used to test the energy label’s influence on the participants’ interest in and attention to energy-related information (i.e., Hypothesis 1 and Hypothesis 6).

#### Mean fixation duration

Mean fixation duration is calculated by dividing the fixation times by the fixation count. This means that it is not directly affected by the amount of information provided to a person, but it is an indicator of the complexity of the integrated information (e.g., [69]). There are no definitive thresholds for the classification of the mean fixation durations [61, 70]. Longer mean fixation durations are associated with more complex information integration, whereas shorter mean fixation durations reflect easier information integration [63, 66, 71]. This parameter was used to probe whether presenting the energy label would facilitate the integration of energy-related information (i.e., Hypothesis 2). Furthermore, this parameter was applied to assess the complexity of energy-efficiency information compared to information about annual consumption (i.e., Hypothesis 5).

#### Number of fixations

Also known as fixation density, fixation count is a frequently used metrics in eye-tracking research, especially in usability and reading research (e.g., [72–74]). Fixations on a certain area suggest that this information is important and noticeable to a person [72]. This means that areas with more fixations receive more attention than those with fewer fixations. Many factors influence where people look; however, the visual features of the material presented and each participant’s intention play important roles [75]. Higher importance is therefore associated with a higher count of fixations [76]. We used this variable to test whether the salient and easily accessible presentation format of energy-efficiency information on the energy label would mislead participants to pay closer attention to this information, whereas the numerical information about annual consumption would attract significantly less attention (i.e., Hypothesis 4).

## Results

### Effect of Energy Label on Availability of Energy-related Information

#### General consideration of energy-related information

Hypothesis 1 stated that presenting the energy labels next to the products would enhance the focus on energy-related information. We used the dwell time for the AOI energy information (see Fig 3 for television and Fig 4 for freezer) as a measurement for the participants’ consideration of energy-related information. Because there were no time restrictions, the decision time differed substantially among subjects (decision time for television in seconds [s]: M [SD] = 68.76 [39.92]; freezer: M [SD] = 64.37 [36.73]. However, there was no significant difference in decision time between the label and the no-label condition in the two focus conditions. There was a significant difference between the decision time for television (M [SD] = 67.65 [43.57]) and freezer (M [SD] = 60.04 [34.91] in the condition energy-saving focus, t (58) = 2.19, p = .033. The difference in the self-focus condition did not reach significance (p = .758). The participants with a longer decision time consequently tended to take a longer dwell time for the AOI energy information and vice versa (television: *r* = .72, p < .01; freezer: *r* = .71, p < .01). To control for these individual differences in decision times, we calculated the relative time by dividing the dwell time for the AOI energy information by the total time needed for decision making in this task [77]. Thus, the relative dwell time reflected the relevance of energy-related information (i.e., measured with the percentage of time that a participant spent on such information during the task). A lower percentage would therefore reflect lower attention (i.e., relevance) toward energy information and vice versa. A similar procedure was used by Ashby and colleagues [78].

**Fig 6:**
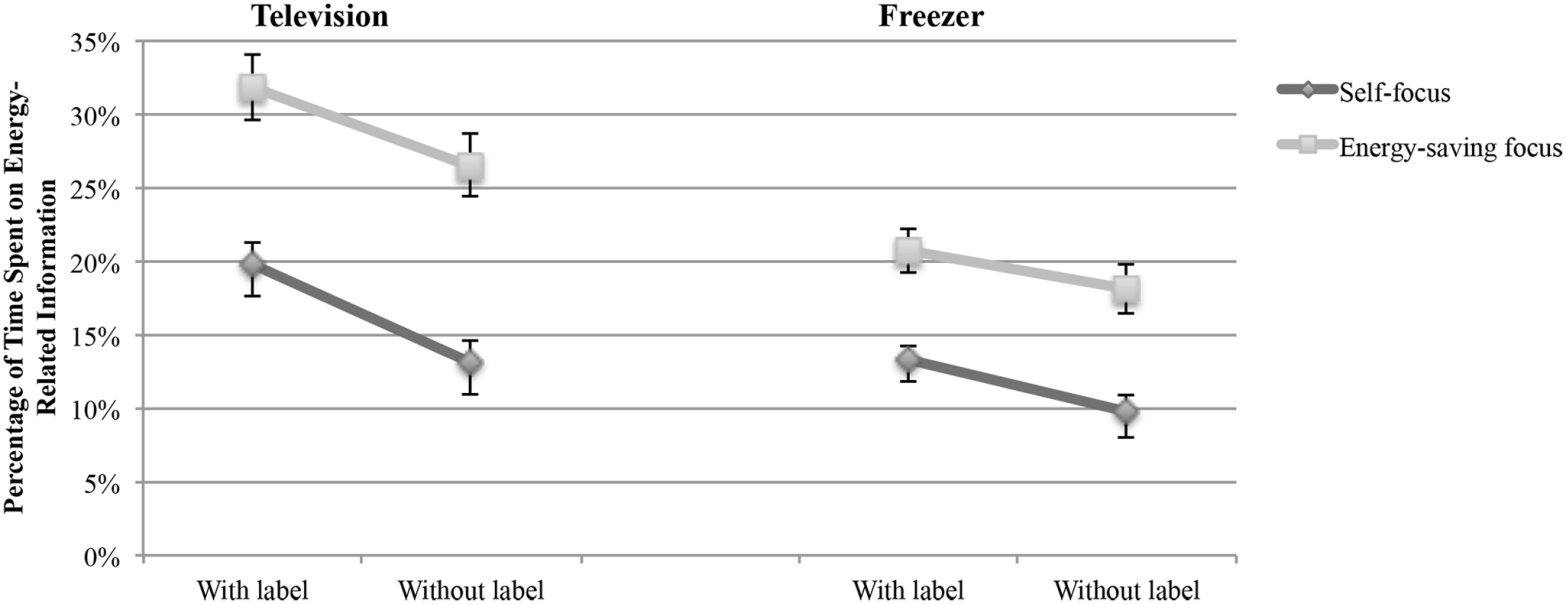
Percentage of decision time spent on energy-related information as a function of label (with vs. without label) and focus (self vs. energy saving). The error bars represent the standard error.

A two-way ANOVA was conducted, with the dependent variable relative dwell time on energy information and the factors label (with vs. without label) and focus (self vs. energy saving) as the independent variables. The results revealed a significant main effect of the factor label on the time spent on energy-related information for the television task, F(1, 113) = 10.61, p < .001, and the freezer task, F(1, 113) = 5.17, p = .025. There was also a significant main effect of the factor focus, F(1, 113) = 46.62, p < .001 (television); F(1, 113) = 34.34, p < .001 (freezer). The interaction of the factors label and focus did not reach the level of significance, F(1, 113) = 0.16, p = .689 (television); F(1, 113) = 0.15, p = .695 (freezer).^2^ The results of the ANOVA are presented in Fig 6. In a further step, a simple main effect analysis of the two factors label and focus was conducted to test for differences in their individual levels [79]. This meant that the effect of the factor focus was separately assessed for each level of the factor label. In this case, the self-focus and the energy-saving focus conditions were separately compared on each level of the factor label (i.e., with label and without label). Likewise, the label and the non-label conditions were separately compared on each level of the factor focus. In line with our hypothesis, the effect of the factor label on the individual levels of the factor focus was significant in the television task, F(1, 113) = 6.63, p = .011 (self-focus) and F(1, 113) = 4.12, p = .045 (energy-saving focus). However, in the freezer task, the effect was non-significant: F(1, 113) = 3.52, p = .063 (self-focus) and F(1, 113) = 1.78, p = .184 (energy-saving focus). This meant that providing the energy label enhanced the relevance and salience of energy-related information in the television task but not in the freezer task.

Furthermore, there was a significant effect of the factor focus on the individual levels of the factor label (i.e., with label vs. without label); television: F(1, 113) = 26.82, p < .001 (without label) and F(1, 113) = 20.13, p < .001 (with label); freezer: F(1, 113) = 20.07, p < .001 (without label) and F(1, 113) = 14.57, p < .001 (with label). This meant that the participants with an energy-saving focus spent more time on energy-related information compared with those with a self-focus (see S1 and S2 Datasets for detailed information about participants’ attention distribution). This result was consistent with Hypothesis 6 and indicated the success of the manipulation.

The findings of the analysis of the absolute time spent on energy-related information were mostly consistent with the results of the relative time. The results revealed a significant main effect of the factor focus for the television task, F(1, 113) = 7.79, p = .006, and the freezer task, F(1, 113) = 5.57, p = .020. The main effect of the factor label was significant in the freezer task, F(1, 113) = 4.18, p = .043, and marginally significant in the television task, F(1, 113) = 3.22, p = .075.

#### Facilitation of integration of energy-related information

According to Hypothesis 2, energy-related information should be more accessible and easier to understand if the energy label was provided as an additional source of information compared to a presentation in a table format only (i.e., condition without the energy label). As stated, mean fixation duration is a parameter used to assess the complexity of integrated information. Shorter mean fixation durations reflect easier information integration, whereas longer mean fixation durations are associated with more complex processes of information integration.

**Fig 7:**
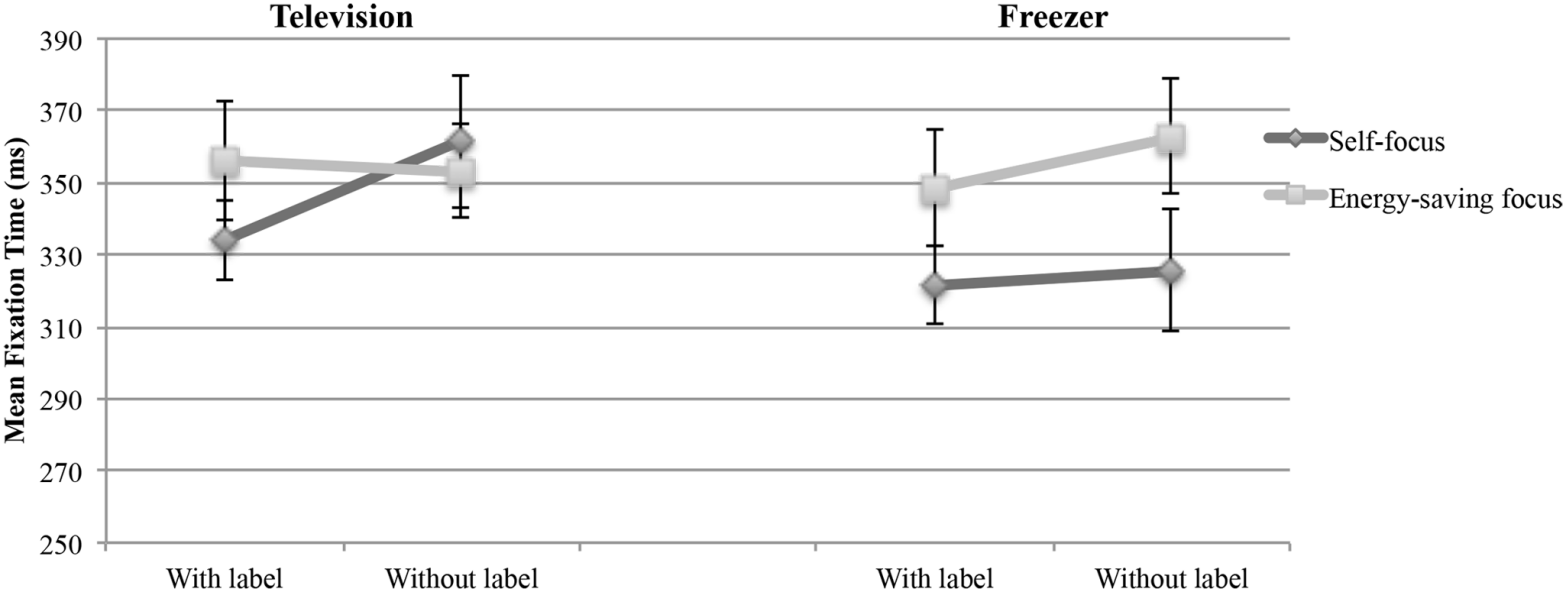
Mean fixation duration on energy information as a function of label (with vs. without label) and focus (self vs. energy saving). The error bars represent the standard error.

A two-way ANOVA was conducted with the factors label and focus as independent variables and the mean fixation duration for energy information as the dependent variable. There were no significant main effects of the factor label, F(1, 110) = 0.66, p = .420 (television); F(1, 111) = 0.37, p = .542 (freezer). The main effect of the factor focus was non-significant for the television, F(1, 110) = 0.21, p = .648, but significant for the freezer, F(1, 111) = 4.51, p = .036. The interaction between the factors focus and label was not significant, F(1, 110) = 1.05, p = .307 (television); F(1, 111) = 0.12, p = .732 (freezer). The results (Fig 7) indicated that energy-related information was not easier to understand if the label was presented to the participants compared with presenting the energy-related information in a table format only. A possible explanation for the significant main effect of the factor focus in the freezer task might be that the participants who chose for themselves were scanning the information rather than integrating it and therefore had shorter mean fixation durations.

#### Energy-friendly Choices

Hypothesis 3 stated that the energy label might alter participants’ choices. In both tasks, one product each was the most energy-friendly choice due to its lowest annual energy consumption—freezer SG and television UE. Subsequently, participants’ choices were categorized as either energy friendly (i.e., freezer SG and television UE) or energy unfriendly to test whether providing the energy label resulted in more energy-friendly product choices. Subsequently, a Chi-square test of independence for the factor choice (i.e., energy friendly vs. not energy friendly) over the four experimental conditions (i.e., self-focus with label, self-focus without label, energy-saving focus with label, and energy-saving focus without label) was calculated. In the television task, a Chi-square test of independence revealed a marginally significant difference between the distributions of observed cases and expected cases in the four experimental conditions, *X* ^*2*^(3, 117) = 6.55, p = .088. For the freezer task, the Chi-square test of independence was significant for the four conditions, *X* ^*2*^(3, 117) = 17.46, p < .001. Table 2 exhibits the choices in the television and the freezer task for each condition. The analysis of the frequencies suggested that the significant effect was due to the factor focus. More precisely, participants with an energy-saving focus chose the energy-friendly product more often compared with participants with a self-focus. A logistic regression with the factors label and focus as predictors on the dependent variable choice supports this result, revealing only the factor focus as a significant predictor for the choice (i.e., energy friendly vs. not energy friendly). However, providing the energy labels as a source of information did not result in a higher number of energy-friendly product choices (consult S1 and S2 Datasets for further information about participants’ attention distribution). There were no effects of sociodemographic variables (e.g., household size) with regard to the choice distribution.

**Table 2:** Choice Frequencies in the Television and the Freezer Task.

Product	Focus	Label	n	Energy-friendly choice	Not energy-friendly choice
Television	Self	yes	28	5	23
		no	30	8	22
	Energy saving	yes	29	14	15
		no	30	10	20
Freezer	Self	yes	28	16	12
		no	30	13	17
	Energy saving	yes	29	24	5
		no	30	26	4

### Energy-Efficiency Class vs. Annual Energy Consumption

#### Relevance of information presentation format

To test Hypothesis 4, we compared the percentages of fixations on the information related to energy efficiency and annual energy consumption, respectively. These variables were computed by summing up the fixations on the AOIs containing the information about energy efficiency and annual consumption, respectively, and dividing this number by the number of all fixations during a task. This procedure resolved the problem of different information loads (i.e., different numbers of AOIs) in the conditions with and without the label.

**Fig 8:**
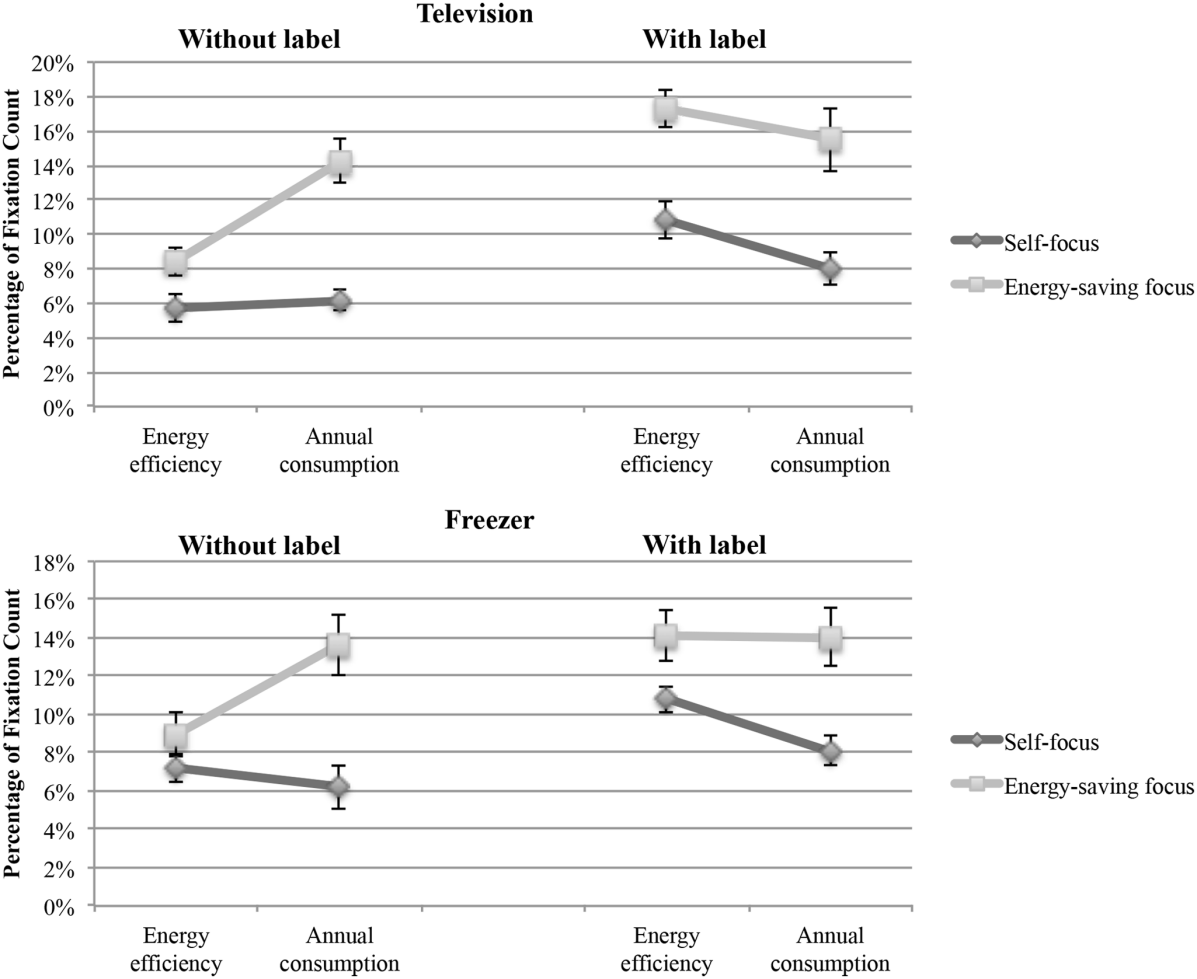
Percentages of fixation count as a function of information format (energy efficiency vs. annual consumption), label (with vs. without label), and focus (self vs. energy saving). The error bars represent the standard error.

First, a mixed ANOVA was conducted with the between-subjects factor focus (self-focus vs. energy-saving focus) and factor label (with vs. without label) and the within-subjects factor information format (energy efficiency vs. annual energy consumption). The count of fixation on these information areas divided by the total count of fixations in all AOIs constituted the dependent variable. The results revealed a significant main effect for label (television: F(1, 113) = 19.43, p < .001; freezer: F(1, 113) = 12.14, p = .001) and for focus (television: F(1, 113) = 41.21, p < .001; freezer: F(1, 113) = 33.65, p < .001). There was a significant interaction information format x focus, F(1, 113) = 5.47, p = .021 (television); F(1, 113) = 5.58, p = .020 (freezer) (see Fig 8). In the television task, there was a significant interaction information format x label, F(1, 113) = 15.76, p < .001. In the freezer task, the interaction did not reach significance, F(1, 113) = 3.43, p = .066. All remaining effects were non-significant (F < 2.56, p > .112). The same analysis was conducted with the absolute fixation count on energy efficiency and annual consumption as dependent variables (i.e., factor information format). The results were mostly consistent with the reported analysis revealing a significant main effect for the factor label (television: F(1, 113) = 5.44, p = .021; freezer: F(1, 113) = 9.93, p = .002) and for the factor focus in the television task, F(1, 113) = 7.92, p = .006. The main effect of focus in the freezer task was marginally significant, F(1, 113) = 3.31, p = .071. The interaction information format x focus was significant for television, F(1, 113) = 9.40, p = .003, and for freezer, F(1, 113) = 10.22, p = .002. The same was true for the interaction information format x label (television: F(1, 113) = 20.66, p < .001; freezer: F(1, 113) = 5.52, p = .021).

To break down the interaction effects of the mixed ANOVA, we conducted a follow-up analysis with paired t-tests for each experimental condition. The results provided additional support for our hypothesis of an energy-efficiency fallacy triggered by the energy label (Table 3). If the participants with an energy-saving focus were not influenced by the energy label (no-label condition), they showed a desired behavior by looking more often at the information about annual consumption, which would be more relevant for assessing a product’s energy friendliness. The participants who chose a product for themselves paid equal attention to both information formats. However, in the condition with the label, the participants with the energy-saving focus abandoned the desired behavior, more precisely, they looked with the same frequency at the information about energy efficiency and annual consumption. Moreover, the participants with a self-focus were driven toward energy-efficiency information, which could result in less energy-friendly purchase decisions (e.g., choosing a bigger television due to a better energy-efficiency rating).

**Table 3:** Fractions of Fixation Count of Information about Energy Efficiency vs. Information about Annual Energy Consumption of Television and Freezer.

				Energy Efficiency	Annual Consumption	
Product	Label	Focus	n	M (SD)	M (SD)	t-test results^1^
Television	yes	Self	28	0.11 (0.06)	0.08 (0.05)	*t*(27) = 1.99, ***p* = .029**, *d* = .376
	yes	Energy saving	29	0.17 (0.09)	0.16 (0.10)	*t(28)* = 0.87, *p* = .197, *d* = .161
	no	Self	30	0.06 (0.04)	0.06 (0.03)	*t(29)* = 0.78, *p* = .234, *d* = .143
	no	Energy saving	30	0.08 (0.04)	0.14 (0.07)	*t(29)* = 5.49, ***p* < .001**, *d* = 1.198
Freezer	yes	Self	28	0.11 (0.04)	0.08 (0.04)	*t*(28) = 3.10, ***p* = .003**, *d* = .584
	yes	Energy saving	29	0.14 (0.07)	0.14 (0.08)	*t(28)* = 0.06, *p* = .477, *d* = .014
	no	Self	30	0.07 (0.04)	0.06 (0.06)	*t(29)* = 0.79, *p* = .218, *d* = .144
	no	Energy saving	30	0.09 (0.06)	0.14 (0.09)	*t(29)* = 2.25, ***p* = .016**, *d* = .440

*Note*. Results of paired t-tests, including means and standard deviations. Significant results are in boldface. Detailed analysis showed that the majority of the participants looked at the information about energy efficiency and annual consumption of each product. Thus, the effect was not due to the data on one product.

^1^One-tailed p-values are indicated.

#### Complexity of energy-related information

We hypothesized that the information about annual energy consumption (kWh) would be more complex than that about energy efficiency (Hypothesis 5). To test Hypothesis 5, mean fixation durations for these two informational attributes were compared. As previously mentioned, longer mean fixation durations reflect more complex information processing, whereas shorter mean fixation durations are associated with easier information integration. The results of a two-way mixed ANOVA revealed a significant main effect of the within-subjects factor information format, F(1, 105) = 26.90, p < .001 (television); F(1, 97) = 44.95, p < .001 (freezer). Additionally, the interaction between the factors information format and label was significant in the television task, F(1, 105) = 5.15, p = .025, but not in the freezer task, F(1, 97) = 1.03, p = .314. The remaining effects were all non-significant (F < 3.18, p > .077).

Table 4 shows the results of the dependent t-tests conducted after investigating the effects in a two-way mixed ANOVA. In all conditions, mean fixation duration was lower for energy efficiency than for annual energy consumption. The difference was significant in all conditions except one (self-focus without the label), indicating that the information about annual energy consumption was more challenging to understand compared to energy-efficiency information. The significant interaction between the factors information format and label in the television task suggested that the label has an influence on the accessibility of information about annual consumption and energy-efficiency information. Thus, the complexity of energy-consumption information and the accessibility of energy-efficiency information might explain why consumers tended to focus more on energy efficiency.

**Table 4:** Mean Fixation Duration (ms) for Information about Energy Efficiency vs. Information about Annual Energy Consumption of Television and Freezer.

				Energy Efficiency	Annual Consumption	
Product	Label	Focus	n	M (SD)	M (SD)	t-test results^1^
Television	yes	Self	27	308.75 (70.70)	357.57 (78.94)	*t*(26) = 2.71, ***p* = .006**, *d* = .523
	yes	Energy saving	28	316.31 (92.22)	403.02 (99.69)	*t(27)* = 5.11, ***p* < .001**, *d* = .965
	no	Self	25	333.19 (79.90)	346.22 (106.76)	*t(24)* = 0.59, *p* = .280, *d* = .118
	no	Energy saving	29	328.09 (82.57)	368.06 (84.63)	*t(28)* = 2.51, ***p* = .009**, *d* = .466
Freezer	yes	Self	27	298.92 (57.69)	341.24 (88.02)	*t(26)* = 2.26, ***p* = .016**, *d* = .435
	yes	Energy saving	27	295.71 (99.64)	387.28 (98.36)	*t(26)* = 3.75, ***p* < .001**, *d* = .684
	no	Self	21	312.28 (72.15)	405.08 (126.45)	*t(20)* = 3.62, ***p* = .001**, *d* = .791
	no	Energy saving	26	309.16 (98.91)	397.88 (99.48)	*t(25)* = 3.57, ***p* < .001**, *d* = .700

*Note*. Results of paired t-tests, including means and standard deviations. Significant results according to the Bonferroni-corrected, dependent t-tests (p < .013) are in boldface. Detailed analysis showed that the majority of the participants looked at the information about energy efficiency and annual consumption of each product. Thus, the effect was not due to the data on one product.

^1^One-tailed p-values are indicated.

## Discussion

This study tested six hypotheses regarding the impact of the energy label and the information provided on it on consumers’ information search and decision-making behavior. We confirm that the energy label increases the focus on energy-related information (Hypothesis 1), especially in the energy-saving focus condition (Hypothesis 6). Additionally, we showed that the energy label leads to a stronger focus on energy-efficiency information (Hypothesis 4), and that this information is easier to integrate (Hypothesis 5). However, the presence of the energy label does not result in more energy-friendly choices (Hypothesis 3) or facilitate the integration of energy-related information (Hypothesis 2).

The energy label’s goal is to inform consumers about the performance of different products in terms of energy friendliness. However, a precondition is that consumers pay attention to the label and more specifically, to the information it provides. This study’s results suggest that the energy label may be able to enhance the focus on energy-related information in general. The mere presence of the energy label triggers the study’s participants to pay more attention to energy-related information. The label can therefore serve as a trigger for energy information, suggesting a higher awareness of environmental considerations. However, this effect can only be found for the television, but not for the freezer. This means that the energy label does not enhance the focus on energy-related information in the freezer task. More research is needed to investigate whether this is also the case with other products and how this issue can be tackled to ensure the energy label’s effectiveness. This is a crucial point because information that is not considered is unlikely to influence the decision-making process [41, 80]. Expressed differently, the consideration of energy-related information may be the first step toward a more sustainable purchase decision. Nonetheless, the energy label’s impact on enhancing an energy-friendly purchase decision seems rather weak; the results revealed no differences in participants’ choices between the label and the no-label conditions. This finding is consistent with those of other studies investigating the impact of energy labels and energy-related information on consumer choices [81, 82]. The results suggest that personal preferences for other attributes (e.g., price and size) are presumably much more important than energy-related information [23]. Therefore, the energy label’s effect may not show up in the final decision, especially because of the participants’ limited selection of only four products, heavily restricting the variance of energy-friendly product choices.

Information provided on labels needs to be salient and accessible [58]. This means that the label should be as simple as possible without losing precision about its meaning. If this condition is fulfilled, labels can be helpful tools to reach consumers and to communicate the information [25, 44, 47, 83–85]. However, this study’s results have shown some important drawbacks of the energy label with regard to information transfer. One vital concern is that the current presentation format of the energy label fails to facilitate the integration of energy-related information. In other words, the participants do not find it easier to understand energy-related information (e.g., kilowatt-hours), with or without the energy label. Furthermore, the information presentation format on the energy label can even lead to biased information search behavior. The results suggest that the energy label influences the participants to pay less attention to actual energy consumption and to focus more on energy-efficiency information. This so-called energy-efficiency fallacy is problematic because the energy-efficiency rating (e.g., A^+^) is relative to the product size and can therefore not be used to compare different-sized products. To find the most energy-friendly product, consumers need to compare the information about actual electricity consumption (e.g., 100 kwh/year). The longer mean fixation durations indicate that actual energy consumption is hard to understand, and more importantly, it is harder to understand than energy-efficiency information. In the condition without the energy label, information complexity has no effect on the participants’ attention distribution. This means that they pay about the same attention to information about energy efficiency and energy consumption. The crucial point is that in the condition with the energy label, information complexity suddenly comes into play, shifting the participants’ equal attention distribution toward energy-efficiency information. These findings indicate that the energy label seems to trigger heuristic information search behavior, that is, reliance on the information that is easier to integrate. A stronger focus on the energy label might thereby boost the energy-efficiency fallacy. Consequently, if the information search is guided toward energy efficiency, it can result in nonoptimal purchase decisions in terms of final energy consumption. The tendency to rely on energy-efficiency information and to neglect actual energy consumption when estimating the energy friendliness of electric goods may further explain why overall energy consumption is still increasing despite advancements in energy efficiency [31]. However, the interaction between the information format (i.e., energy efficiency vs. annual consumption) and the label is not significant in the freezer task, indicating that the impact of the misleading effect of energy efficiency varies between product types. This means that although the energy label is generic for all product types, its effect on consumers’ decision making depends on the specific product type.

The detected preference for energy efficiency is in line with the findings of various studies (e.g., [23, 31, 45]). The present study’s result suggests that one reason for this consumer behavior (i.e., disregard for actual energy consumption) may be due to the complexity of the information format. The findings are consistent with those of other studies showing consumers’ scant awareness of the actual energy consumption of electric goods [86] and their struggles with the interpretation of technological terms [46].

### Implications

Several implications for policymakers can be derived from the presented results. The promotion of energy efficiency and the implementation of policy tools, such as the energy label, seem to be less efficient than expected. Hence, the mandatory labeling policy is insufficient to enhance sustainable energy consumption. Other policy measures may be needed to successfully reach the energy-saving goals. Furthermore, information presentation formats on labels triggering heuristic thinking can be helpful [41]; however, if the basis for the decision is ambiguous, heuristics can result in a biased decision [53]. The problem lies in the rating system of energy efficiency that does not allow an overall assessment of a product’s performance in terms of energy friendliness. Therefore, it seems worthwhile to consider a rating system that allows the comparison of different-sized products. Consequently, consumers can be sure that if they choose the best-rated product, it is in fact the one with the least consumption.

To further overcome the energy-efficiency fallacy, new solutions for communicating information about actual energy consumption should be considered. In the current communication format, information about annual consumption is harder to understand and less prominent compared to energy-efficiency information. A possible reason is that energy efficiency is communicated with a pictogram (i.e., letter scale and colored) [44]. This means that processing energy consumption information should be facilitated and must become more accessible and salient on the energy label. For example, a study in the tourism sector has found that the combination of color and factual information facilitates comprehensibility [84]. Adding a graphic cue for an appliance’s performance that is based on its actual consumption, compared to those of all other appliances, can be beneficial for consumers [87]. Moreover, the energy consumption of an average appliance can be added to provide a reference point for the kilowatt-hour number. Facilitating information about energy consumption could help neutralize the effect of the energy-efficiency fallacy.

### Limitations and Future Research Directions

A number of limitations need to be kept in mind when interpreting the results presented in this study. Although the analysis was based on an objective measurement method (i.e., eye tracking), the results were retrieved from a simulated experiment; thus, it might only partially reflect real-life and consumer decisions, respectively. For example, the participants might have paid more attention to actual energy-consumption information because they might have felt obligated to study the materials presented more carefully, due to the simulated setting, than they would in reality. Furthermore, the presentation design of the material had to be optimized for the eye tracker, which means that it differs from the presentation design of existing online shops and is somewhat artificial. However, many online shops provide a selection of products for direct comparison that is comparable to the presentation design in the study. Additionally, all relevant energy-related information was depicted in the experiment, whereas in real life, some information––in most cases, about actual energy consumption––is often missing. The observed information search bias toward energy efficiency might therefore even be stronger when consumers would be in a real purchase situation.

The validity of eye-tracking data has been proven in many studies in various fields (for an overview, see [57, 88]) and it provides an objective measurement of participants’ gaze behavior. However, the data needs interpretation and is therefore never absolutely conclusive. Furthermore, we could not rule out that the stimulus material itself had an impact on participants’ viewing behavior. Although the participants were randomly assigned to the experimental conditions, certain aspects of the stimulus material, such as the saliency of the different pictures of the products or the colorful energy labels, might have affected the results. Moreover, the participants’ physical constitution (e.g., tired), interest, and motivation might be influential factors that could not be absolutely controlled for. For example, freezers were probably of less interest to the participants than televisions (e.g., due to the latter’s more technical features and higher status symbol) [89]. This perception of product categories is consistent with findings of previously conducted qualitative interviews conducted by the authors of this paper with consumers who had just bought an electric good. A reduced interest in freezers (i.e., reduced cognitive effort) could explain why the participants were rather scanning energy-related information in the freezer task [63]. Information complexity also provides an adequate explanation for participants’ gaze behavior or more precisely, for the energy-efficiency fallacy. Additional research should be conducted to study which information presentation format can help overcome the fallacy and lead to unbiased information search behavior.

Furthermore, this study was not designed to investigate consumers’ final decisions but the process (i.e., information search and decision-making behavior) that would eventually lead to the final decision. Hence, more research is needed to verify the rather low impact of the energy label on the purchase decisions detected in this study. For example, field or conjoint-based experiments could reveal the importance of the energy label for consumers’ product choices in a more sensitive way. In assessing the impact of the misleading effect of the energy label (i.e., energy-efficiency fallacy) on product choice, no final conclusion could be drawn from the results presented here because the choice was binary with regard to energy friendliness (i.e., energy friendly vs. not energy friendly). More precisely, to assess the fallacy’s impact on consumer decisions, a more sensitive measurement would be needed to detect differences in consumer choices. Further research could investigate the extent to which consumers would be misled by the energy label.

Finally, this study concentrated on a typical consumer product (i.e., television) and a typical household appliance (i.e., freezer) because it was not feasible to test all product types with a labeling obligation. The results revealed differences between the two product types regarding the energy label’s impact. Further research should investigate to what extent the presented findings can be generalized to other products. Furthermore, the detected differences between products reinforce the importance of including various products when evaluating the energy label.

## Supporting Information

S1 Dataset Count of fixations on all areas of interest in the condition without the energy label. (SAV)

S2 Dataset Count of fixations on all areas of interest in the condition with the energy label. (SAV)

S1 Fig Products with pictures and freezer features in the label condition. (TIF)

S2 Fig Products with pictures and freezer features in the no-label condition. (TIF)
