# Supplementary figures and images for: Desired and Undesired Effects of Energy Labels—An Eye-Tracking Study
Source: PLoS One. 2015 Jul 31;10(7):e0134132. doi: 10.1371/journal.pone.0134132 (PMC4521693; doi:10.1371/journal.pone.0134132)

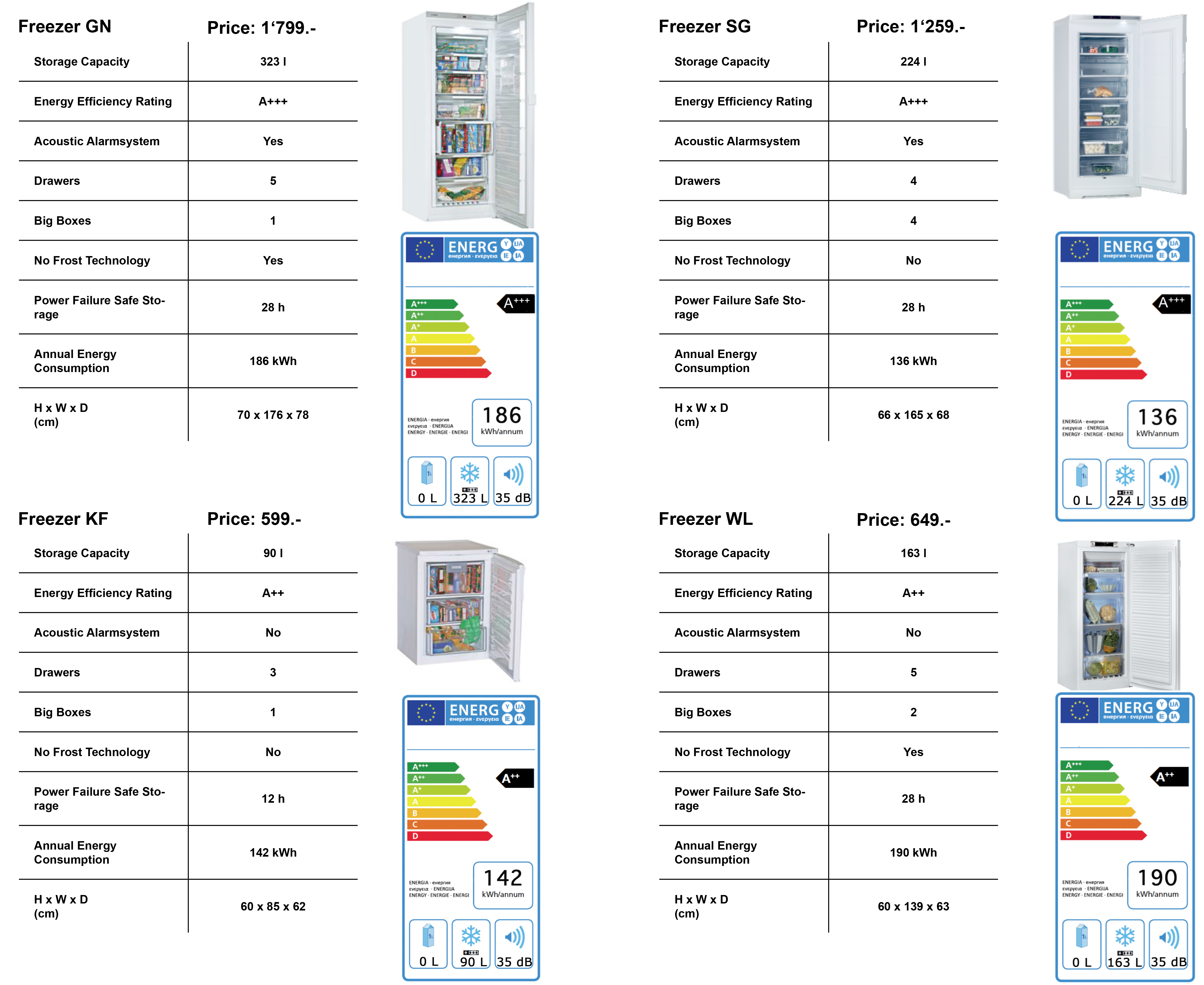

Supplement: S1 Fig — (TIF) [file pone.0134132.s003.tif]

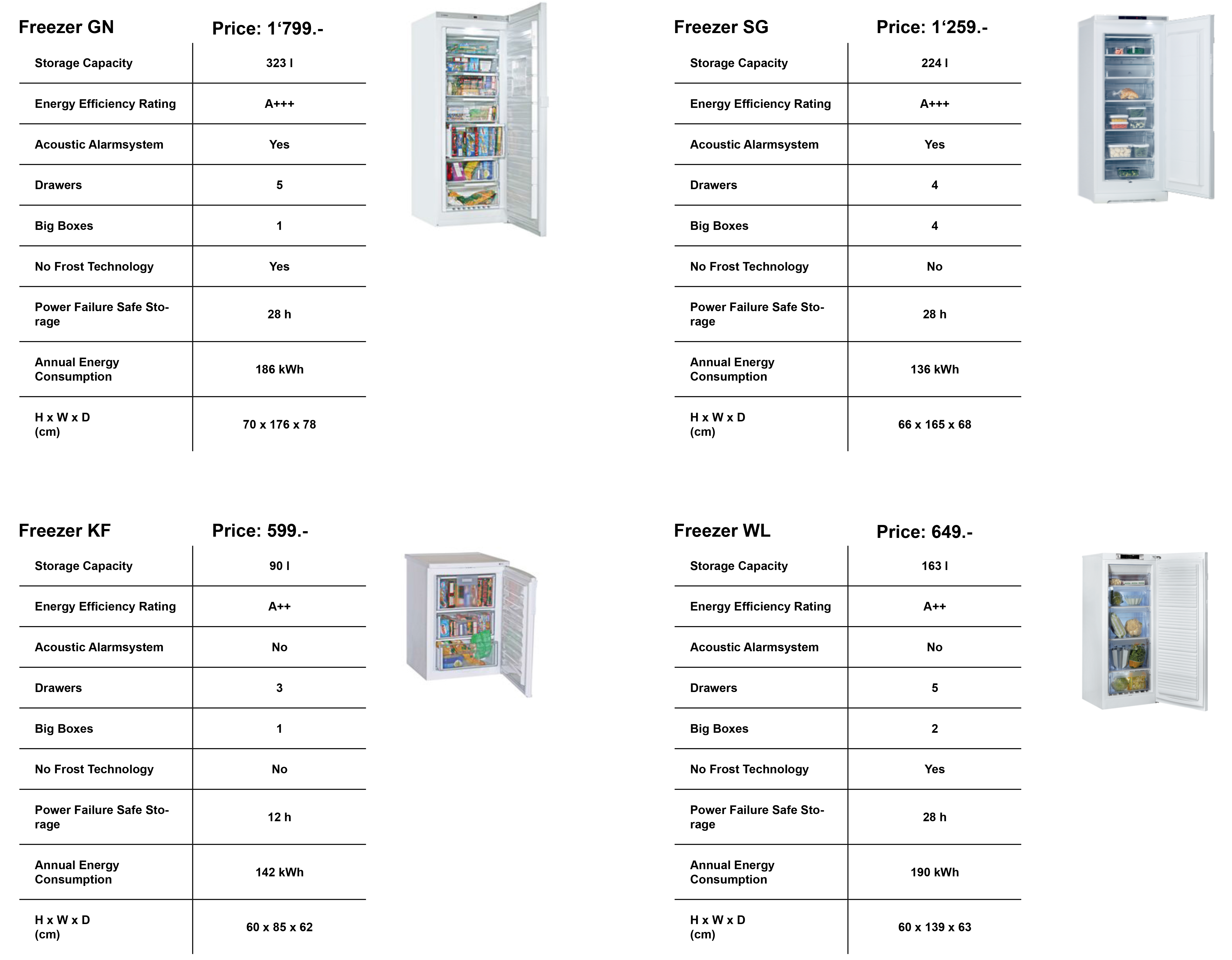

Supplement: S2 Fig — (TIF) [file pone.0134132.s004.tif]
